# Convergent evolution of giant size in eurypterids

**DOI:** 10.1098/rspb.2024.1184

**Published:** 2024-07-31

**Authors:** Alexander Ruebenstahl, Nicolás Mongiardino Koch, James C. Lamsdell, Derek E. G. Briggs

**Affiliations:** ^1^ Department of Earth and Planetary Sciences, Yale University, New Haven, CT 06520, USA; ^2^ Scripps Institution of Oceanography, UC San Diego, La Jolla, CA 92093, USA; ^3^ Department of Geology and Geography, West Virginia University, 98 Beechurst Avenue, Brooks Hall, Morgantown, WV 26506, USA; ^4^ Yale Peabody Museum, Yale University, New Haven, CT 06520, USA

**Keywords:** arthropod, palaeozoic, sea scorpion, gigantism, predator, convergence

## Abstract

Eurypterids—Palaeozoic marine and freshwater arthropods commonly known as sea scorpions—repeatedly evolved to remarkable sizes (over 0.5 m in length) and colonized continental aquatic habitats multiple times. We compiled data on the majority of eurypterid species and explored several previously proposed explanations for the evolution of giant size in the group, including the potential role of habitat, sea surface temperature and dissolved sea surface oxygen levels, using a phylogenetic comparative approach with a new tip-dated tree. There is no compelling evidence that the evolution of giant size was driven by temperature or oxygen levels, nor that it was coupled with the invasion of continental aquatic environments, latitude or local faunal diversity. Eurypterid body size evolution is best characterized by rapid bursts of change that occurred independently of habitat or environmental conditions. Intrinsic factors played a major role in determining the convergent origin of gigantism in eurypterids.

## 1. Introduction

**Figure 1.**
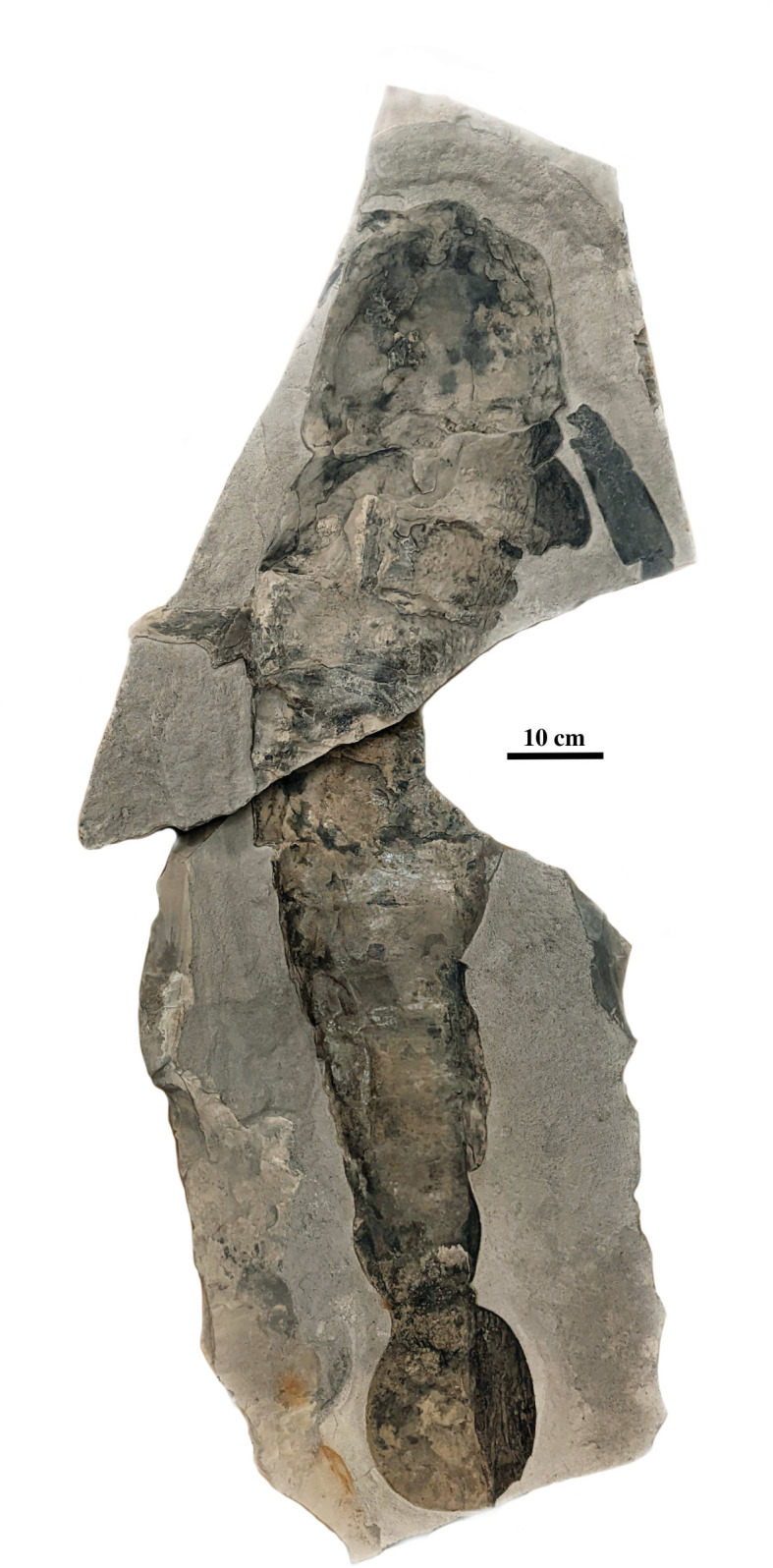
The giant eurypterid *A. macrophthalmus*. A giant specimen of the pterygotid eurypterid *A. macrophthalmus* about 1.25 m long from the Fiddlers Green Formation at Passage Gulf in upper New York State. This specimen (Yale Peabody Museum IP 208195) is one of the largest known complete examples of a eurypterid, if not the largest. The length of eurypterids over 2 m long is based on extrapolation from the dimensions of isolated elements of the exoskeleton.

Eurypterids, commonly known as sea scorpions, are extinct chelicerates that ranged from the Ordovician to the late Permian [1]. They are renowned as giant predators and repeatedly evolved body lengths in excess of 1 m, excluding their chelicerae. Eurypterids comprised two major clades, the paddle-limbed eurypterines and the long-limbed stylonurines, both of which inhabited marine to continental (freshwater) settings [2,3] and are known from all continents except Antarctica [4]. The smallest eurypterids were less than 2 cm long as adults [5], whereas the largest exceeded 2.5 m [6]. Seven of the 21 recognized families include representatives more than a metre in length, such as *Acutiramus macrophthalmus* (figure 1) [3,4]. Giant eurypterids tend to be deeply nested within major clades reflecting multiple instances of convergent evolution to giant size.

Despite repeated evolution to some of the largest body sizes reached by arthropods, the factors that led to giant size in eurypterids have not been analysed comprehensively [6]. The classic hypothesis that competition with gnathostomes (jawed fishes) drove the evolution of giant eurypterids [7] lacks support [8,9] although it is still invoked [10]. Gigantism among other Palaeozoic arthropods, such as Carboniferous insects and myriapods [11,12], has been attributed to high oxygen levels [13]. The only previous quantitative analysis of eurypterid body size (based on a limited sample of eurypterines [14]) found no evidence that large (>0.5 m) or giant (>1 m) body size is a response to atmospheric oxygen, sea surface temperature or sea level change, nor that it occurs at higher latitudes, as implied by Bergmann’s rule (1847) [15] relating larger size to lower temperatures at high latitudes. Later Palaeozoic stylonurines were largely freshwater inhabitants and capable of brief terrestrial excursions [16,17] and many reached lengths of over a metre. Living chelicerates have a haemolymph with a similar ionic concentration to sea water [18], and eurypterids may have faced issues with osmoregulation when transitioning from euryhaline to freshwater habitats. Some extant terrestrial crustaceans reach enormous sizes to avoid desiccation [19] and it has been hypothesized that giant size in eurypterids may be related to habitat shifts [16].

There is no consensus on which of several factors provide an explanation for gigantism in eurypterids, a trait that characterizes a niche of apex nektonic arthropod predators that is absent in modern ecosystems. Clearly, the acquisition of giant size multiple times across deep time requires analysis within a phylogenetic framework. We assembled a large dataset of maximum size and extrinsic factors (electronic supplementary material, dataset 1) and conducted the first phylogenetically informed analysis of the evolution of eurypterid body size, testing whether palaeotemperature, oxygen availability, habitat shifts or mass extinctions drove its macroevolutionary dynamics. We also considered other factors that have been proposed as possible drivers of gigantism in eurypterids—palaeolatitude and community composition, including competition.

## 2. Material and methods

Maximum sizes were sourced from Lamsdell and Braddy [3], combined with information on newly described taxa [1] and new data on maximum size [20]. The 138 species included (electronic supplementary material, dataset 1) represent a sample of nearly all valid and relatively completely known eurypterids.

We assembled a dataset of sea surface temperature, levels of dissolved O_2_ and depositional environment (electronic supplementary material, dataset 1). Data were also compiled for a preliminary investigation of possible correlations with the taxonomic diversity of assemblages (co-occurring eurypterids and other taxa, including potential competitors) in which eurypterids occur (electronic supplementary material, figure S7), and for latitude (electronic supplementary material, figure S8). The data, apart from sea surface temperature and levels of dissolved O_2_, were extracted from the Paleobiology Database (PBDB; paleobiodb.org—accessed September 2022) updated, as necessary, with information from recent literature (Dataset 1). We explored the relationship between the various factors and eurypterid size in the context of an updated phylogeny based on the morphological matrix of Lamsdell and Selden [9], including 226 characters and 154 taxa (electronic supplementary material, figure S1). Tip-dated Bayesian inference under the fossilized birth-death (FBD) process was carried out in MrBayes v. 3.2.7 [21,22]. Stratigraphic ranges were used as uniform priors on tip ages, and an uncorrelated relaxed morphological clock was enforced [23]. Morphological change was modelled using a Mk_v_ + Г model [24], partitioning characters by their number of states, and including an ascertainment bias correction for coding only variable characters. After a rapid diversification, eurypterid diversity declined sharply during the Devonian Biotic Crisis [3] owing to decreased speciation rates and habitat fragmentation. We incorporated this knowledge into the inference procedure through a skyline FBD tree prior [25,26] including three successive diversification/preservation regimes (representing radiation, extinction and subsistence phases). For further discussion of phylogenetic methods see electronic supplementary material, text 1. Inference was summarized using both a maximum clade credibility (mcc) tree and 500 randomly sampled posterior trees. Trees were pruned to the set of ingroup terminals (135) with information on body size and depositional environment.

Palaeozoic sea surface temperatures are the subject of continued debate [27–29]. We used the estimates of Song *et al*. [30], who focused on lower latitudes and inferred less extreme early Palaeozoic temperatures than other authors [29], although the patterns are similar. Temperatures were reported as averaged values spanning 1 Myr intervals. Dissolved ocean surface oxygen values were derived from the temperature estimates ([30], fig. 5). Estimates of sea surface dissolved oxygen were preferred to atmospheric oxygen as the majority of eurypterids were marine [17,31]. Later examples were capable of limited terrestrial mobility with the aid of respiratory structures called *Kiemenplatten* [17] and pillar-like lung trabeculae [31]. Values were digitized to obtain estimates every 1 Myr using WebPlotDigitizer v. 4.6 [32]. Natural smoothing splines were then fit to both and interpolated to 0.25 Myr intervals.

Depositional environments were divided into three categories: (i) marine (reefs or definite offshore deposits); (2) marginal marine (shallow protected settings such as bays and estuaries); and (iii) continental (lakes and rivers). Taxa that occur in multiple formations were coded based on the formation for which the most complete data are available (completeness of fossils, abundance and confidence of assignment; electronic supplementary material, dataset 1, see Notes column). Most fossils are exuviae and some eurypterids may be preserved in marginal marine environments that they did not normally occupy [2,33]. It is unlikely, however, that they moved between marine and fresh water to moult. Environments were inferred, in some cases, based on lithology (electronic supplementary material, dataset 1, see Notes column). The evolution of this trait was explored using reverse-jump Markov chain Monte Carlo (rjMCMC) in BayesTraits v. 3.0.1 [34], stochastic character mapping [35] and state-dependent lineage-through-time plots [36].

Eurypterid body size was log-transformed and its macroevolutionary dynamics evaluated using 11 models. These can be subdivided into approaches that attempt to characterize morphological change without invoking extrinsic factors, and approaches designed to test putative drivers of eurypterid gigantism. Among the first, we explored a Brownian motion model (i.e. a random walk with constant variance) as well as extensions of this framework that incorporate constrained evolution within an adaptive zone (Ornstein–Uhlenbeck model, OU), active trends, and exponentially varying rates that allow for early burst dynamics. All of these models are intrinsically gradualistic, with expected outcomes that are a function of the amount of time elapsed (branch lengths). Two alternatives that relax this assumption were also explored: a kappa model [37] that allows for divergence to accrue faster than expected (0 < *Κ* < 1), or even independently of phylogenetic branch lengths (*Κ* = 0); and pulsed models [38] that incorporate sudden bursts of change.

Alternatives incorporating extrinsic drivers were implemented using OU models. Body size evolution was coupled with sea surface temperature and dissolved oxygen using non-stationary optima that are linearly dependent on reconstructed curves [39]. Multipeak OU (OUM) models that depict evolution on complex adaptive landscapes were used to test for an effect of ecological transitions (using depositional environment as proxy) and extinction events (Devonian biotic crisis) on body size evolution. In the first case, ecology was mapped using stochastic character mapping under an optimal model of evolution, and ecological regimes were treated as evolving towards distinct adaptive peaks (allowing rates of change and strengths of selection to also vary). Three-peak (marine, marginal marine and continental) and two-peak (marginal marine and other) OUM alternatives were considered, as previous hypotheses suggest gigantism could have been favoured in both continental and fully marine habitats (albeit for different reasons), and a simpler model might efficiently capture this with fewer parameters. A three-peak OUM model was also implemented to assess the impact of the Devonian biotic crisis on body size evolution, with a single peak encompassing all lineages predating the extinction event, and two separate ones for the clades surviving and diversifying in its aftermath. Models were fit to the set of posterior trees, accounting for topological and temporal uncertainties, and ranked using median-corrected Akaike information criterion (AICc) weights as estimates of model fit. The process was repeated separately for Eurypterina and Stylonurina subtrees. Further details on model fitting are provided in electronic supplementary material, text 2. All macroevolutionary analyses were performed in the R environment 4.2.2 [40] using functions from a range of phylogenetic packages [41–44].

Palaeolatitude, also obtained from the PBDB, was modelled as a predictor of body size using phylogenetic regressions (function ‘pgls’ of package caper [45]) on all 500 posterior topologies, constituting a phylogenetic test of Bergmann’s rule [15]. Community composition was investigated by tallying the number of potential predator and prey species for each eurypterid taxon, along with the total number of eurypterid species and the overall species richness of the environments they inhabited. Eurypterid species were split into two groups using a 0.5 m threshold of body size. A permutational ANOVA [46] (1000 replicates) was used to detect significant differences in ecospace occupancy between groups. We acknowledge the overall difficulty in testing these hypotheses in a manner that is devoid of biases (e.g. taphonomic, uneven preservation and sampling), and note where these may have played a determinant role.

## 3. Results

### (a) Distribution of giant size

**Figure 2.**
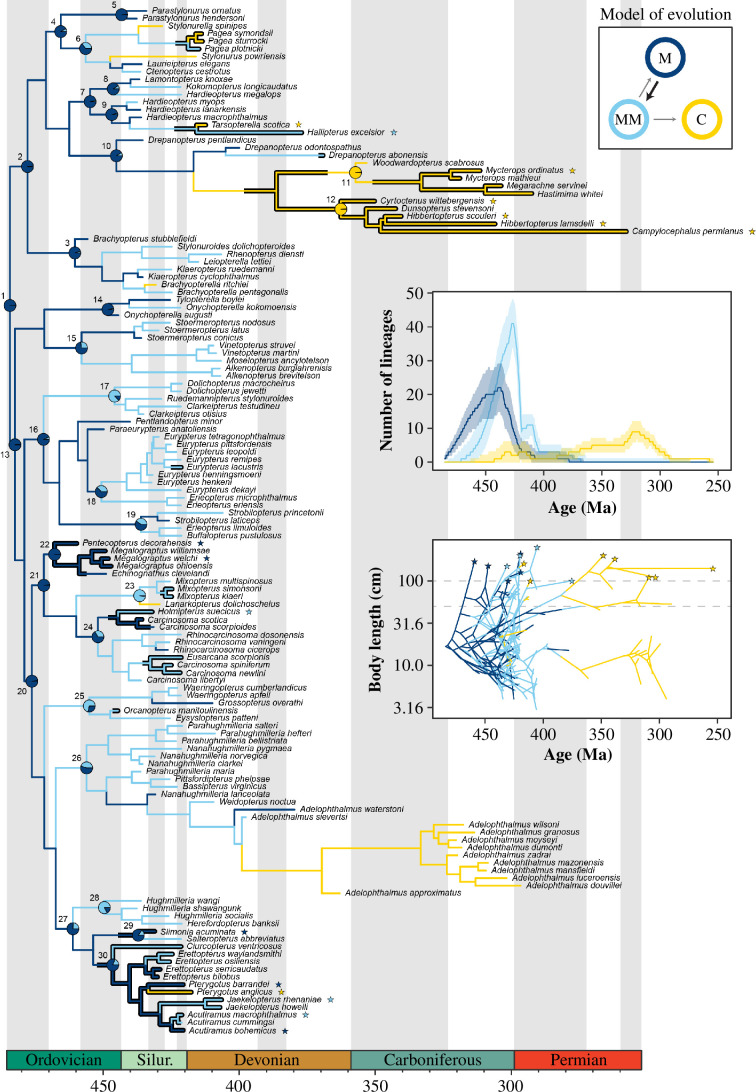
Evolution of eurypterid size and habitat. Phylogenetic relationships (mcc tree) of eurypterids and evolution of body size and habitats: dark blue—marine habitat, light blue—marginal marine, yellow—(continental) freshwater. Favoured model of evolution (upper right) shows relative frequency of transitions between habitats. Centre right inset plot shows diversity in marine, marginal marine and continental (freshwater) habitats across time, obtained using stochastic mappings across posterior topologies (summarized using median and 95% confidence bands). Bottom right inset plot is a phenogram showing body size evolution in the mcc tree, with branches coloured to reflect habitat. Dashed lines denote large (≥50 cm) and giant body sizes (≥100). Pie charts on the main tree show ancestral state reconstruction of habitat for major clades (listed below), summarized from 1000 replicates of stochastic character mapping. The most frequent state is also shown using branch colours. Wider branches indicate large bodied clades; giant terminals are highlighted using stars. Higher taxonomic ranks: 1. Eurypterida, 2. Stylonurina, 3. Rhenopteridae, 4. Stylonuroidea, 5. Parastylonuridae, 6. Stylonuridae, 7. Kokomopteroidea, 8. Kokomopteridae, 9. Hardieopteridae, 10. Mycteropoidea, 11. Mycteropidae, 12. Hibbertopteridae, 13. Eurypterina, 14. Onychopterellidae, 15. Moselopteridae, 16. Eurypteroidea, 17. Dolichopteridae, 18. Eurypteridae, 19. Strobilopteridae, 20. Diploperculata, 21. Carcinosomatoidea, 22. Megalograptidae, 23. Mixopteridae, 24. Carcinosomatidae, 25. Waeringopteroidea, 26. Adelophthalmoidea, 27. Pterygotoidea, 28. Hughmilleriidae, 29. Slimoniidae, 30. Pterygotidae.

Eurypterid relationships inferred under a tip-dated FBD framework (figure 2; electronic supplementary material text 1, figures S1–S3) are largely congruent with those based on previous analyses [9]. No major topological effect was induced by tip-dating, nor by implementing a skyline FBD model with multiple diversification epochs (electronic supplementary material, figure S2). The common ancestor of all eurypterids, i.e. the node that gave rise to the major clades Stylonurina and Eurypterina, was relatively small at about 16.7 cm long (95% confidence interval (CI) = 6.1–45.8 cm). Large size (≥0.5 m) evolved independently 11–18 times (depending on the tree used; 13 times in the mcc tree), and many of those clades contain giant taxa ≥1.0 m (figure 2). Gigantism evolved three times in stylonurines—twice among continental (freshwater) mycteropoids and once in hardieopterids. It was also attained repeatedly by eurypterines (figure 2)—evolving among carcinosomatids, megalograptids and the characteristically large pterygotids. Gigantism originated within clades inhabiting marine, marginal marine and terrestrial environments.

### (b) Sea surface temperature and dissolved oxygen

**Figure 3.**
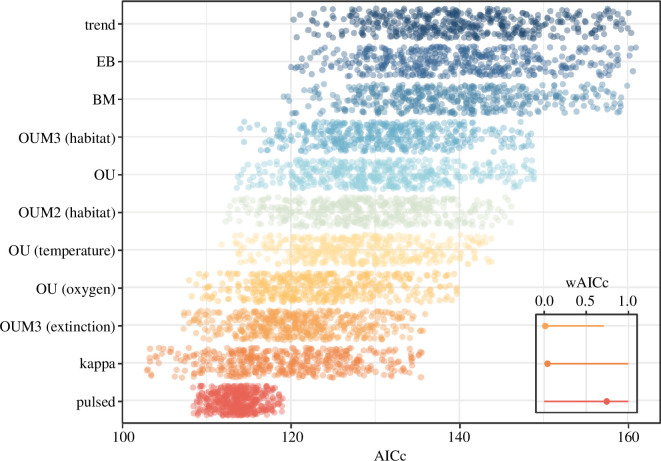
Models of eurypterid macroevolution. Fit of 11 models characterizing the macroevolution of eurypterid body size (AICc = sample size corrected Akaike information criterion scores; wAICc = AICc weights). Dots represent values obtained from each posterior tree, values outside the 95% CI of each model are not shown. Inset shows the median and range of wAICc for the three best-fitting models. Support for a pulsed model dominates (median wAICc = 0.763).

**Figure 4.**
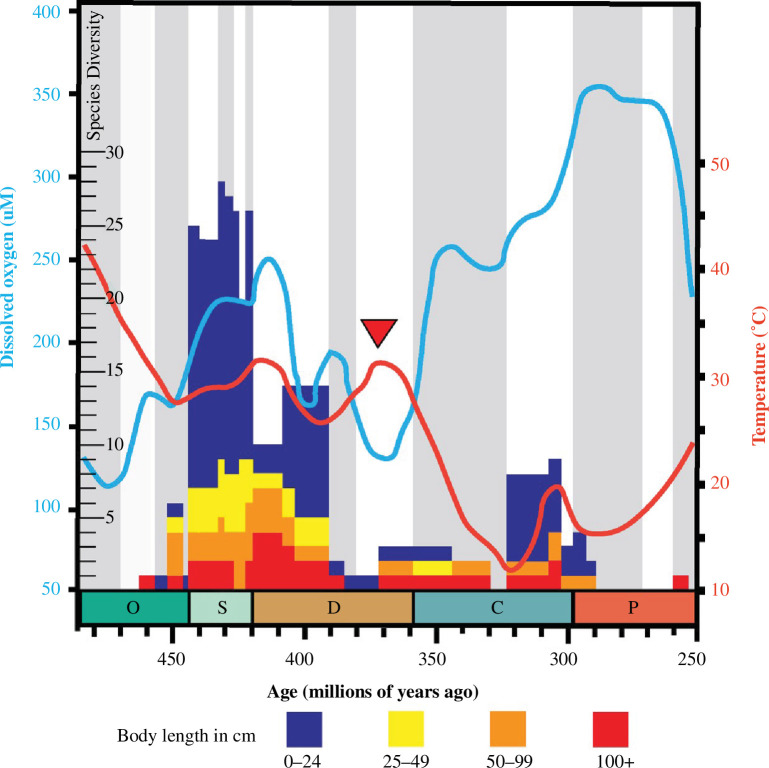
Eurypterid diversity and sea surface dissolved oxygen and temperature. Eurypterid species diversity (stratigraphic occurrences partitioned by body size) through time compared to dissolved sea surface O_2_ (blue) and sea surface temperature (orange). Red arrow indicates the Famennian mass extinction.

We find no relationship between changes in sea surface water temperature or dissolved oxygen levels and the evolution of body size in eurypterids as a whole (figure 3). Oxygen levels appear to have been a factor in the evolution of larger sizes in stylonurines, however, when they are analysed independently of eurypterines (electronic supplementary material, figure S4). This result is driven by continental hibbertopterids and mycteropids that reached giant sizes during higher oxygen levels in the Carboniferous (figure 4; electronic supplementary material, dataset 1). Our oxygen data are based on dissolved sea surface oxygen, but they reflect levels of oxygenation in the atmosphere and presumably fresh water. However, all other eurypterid clades that reached large sizes did so during the Ordovician and early Devonian [1,6], when oxygen levels were much lower. The lack of a universal effect of oxygen levels suggests that the relationship detected within stylonurines might not be causal, and instead emerge from the contingent survival of only large bodied continental stylonurines through the Devonian biotic crisis.

### (c) Habitat shifts

The initial radiation of eurypterids happened entirely in the marine realm (posterior probability (pp) at the tree root = 0.996), and eurypterids colonized continental (freshwater) habitats between 7 and 15 times (nine in the mcc tree; figure 2). The model with the highest pp in the rjMCMC analysis (electronic supplementary material, figure S5), as well as that preferred for the mcc tree (figure 2), implies terrestrialization was only possible through marginal marine environments. Colonizations of continental habitats before the Middle Devonian were ephemeral and did not result in lineage diversification (figure 2 insets). Two significant radiations in continental habitats occurred later, in mycteropids (Stylonurina) and adelophthalmids (Eurypterina), which together represent the entirety of eurypterid diversity by the Carboniferous (figure 2).

Continental stylonurines and eurypterines varied in size from under 10 cm (*Brachyopterella*, *Adelophthalmus duponti*) to over 1.5 m (*Hibbertopterus*, *Pterygotus anglicus*), a range comparable to that in both marine and marginal marine species (figure 2 inset; electronic supplementary material, dataset 1). Although both continental and fully marine taxa were slightly larger on average than those in marginal marine environments (electronic supplementary material, figure S6), this difference is not significant. In fact, we find no evidence that body size evolution was driven by habitat preferences (figure 3). Neither is any direct effect of the Devonian biotic crisis detected (figure 3).

### (d) Other factors

We tested for a correlation between eurypterid size and palaeolatitude [15]. Latitude might influence size indirectly because colder water accommodates more dissolved oxygen and temperature may also impact continental forms [47–50]. Our analysis revealed no clear relationship: *R* ^2^ and slope values were all close to zero and revealed no evidence of a role for climate in influencing size in marine or continental eurypterids [15]. It should be noted, however, that this analysis does not take into account that the overall climate at a given latitude varied considerably with time within the timeframe explored. A previous investigation of the role of palaeolatitude, although it considered only marine taxa and not in a phylogenetic context, obtained the same result [14]. Sampling bias in favour of North America and Europe [4] may play a role. The majority of eurypterids are known from lower palaeolatitudes and large eurypterids are essentially confined to the palaeotropics until the Carboniferous—giant stylonurines were present at higher latitudes as in the Permian (electronic supplementary material, figure S8).

We assembled data on numbers of eurypterid species within communities because greater niche space may favour a larger range in body size [51–56]. Conversely a higher density of closely related species may constrain body size [57]. We also considered total faunal diversity in association with eurypterids as this too may correlate with larger ranges in body size [54]. We found no evidence of correlation in either case.

We used the number of predator and prey species associated with eurypterids as a test of Romer’s hypothesis [7] that competition with jawed gnathostomes was a driver of giant size in eurypterids and a factor in their extinction. Predators were identified as any carnivorous gnathostome fish, nautiloid or eurypterine over 2× the maximum length of a potential prey eurypterid. We did not account for the possibility that smaller juvenile stages were more vulnerable to predation than adults. Prey species were identified as any mobile benthic or nektonic animal less than 50% of the maximum length of a predatory eurypterid. Stylonurines are known to have been sweep feeders [58] and thus were only included as predators if they exceed the body length of a potential prey species by 5×. Hibbertopterids were excluded as they were specialized suspension feeders [58,59]. Reef building and other sessile taxa are not considered to have been eurypterid prey [60]. Taxa from single localities were grouped with those from environmentally similar coeval units. We acknowledge, however, that eurypterids may have lived in different environments to those in which they are preserved and may have encountered a more diverse range of organisms. We found no correlation between the evolution of giant size and the diversity of predator or prey species (electronic supplementary material, figure S7). These results are tentative, however, owing to low sample numbers of gnathostomes (electronic supplementary material, dataset 1). Furthermore, habitats such as the Bertie Waterlime may represent safe refuges for eurypterid moulting [2,33] with fewer predators than in their normal habitat.

### (e) Pulsed evolution of body size

The evolution of body size in eurypterids is best explained by pulsed and kappa models, both of which capture punctuational and non-gradualistic dynamics [61,62] (see §2; figure 3; electronic supplementary material, text 1 and figure S4). Phenotypic bursts or pulses resulted in rapid divergence (figure 2 inset) that allowed numerous lineages to repeatedly access regions of large-bodied and gigantic morphospace at near-instantaneous macroevolutionary timescales, and independently of oxygen levels, palaeotemperature and environment. The average estimate of *Κ* was 0.7 (95% CI = 0.56–0.84), indicative of a process that departs from a pure punctuational process where phenotypic jumps occur only at cladogenetic events [62]. This is further supported by the even better fit of pulsed models that make no such assumption.

## 4. Discussion

### (a) Oxygen

Increase in the body size of metazoans, including terrestrial arthropods, has been shown to relate to oxygen levels [13,63–65] but this relationship is complex in marine and terrestrial arthropods [66,67] and may not apply to eurypterids. Review of our data does, however, suggest a potential relationship between higher oxygen levels and lower sea surface temperatures and higher eurypterid species diversity (figure 4). The increase in diversity following the appearance of eurypterids in the Middle Ordovician (Darriwilian) roughly parallels the rise in ocean surface dissolved oxygen from about 170 µM in the late Ordovician to about 230 µM by the end of the Silurian [30]. Rising oxygen has been invoked as a driver of the Great Ordovician Biodiversification Event [68]. The decline in dissolved sea surface oxygen levels to 140 μM during the late Devonian [30] may have contributed to the Famennian mass extinction [69–71] and impacted the diversity of marine eurypterids. The increase in diversity of continental eurypterids in the Carboniferous coincides with a rise in sea surface oxygen [30] but their subsequent decline occurs when oxygen levels are still rising (figure 4). The possible relationship between oxygen levels and diversity is not the focus of this study, but may prove to be an interesting avenue for future research.

### (b) Transition to continental habitats and diversification

Eurypterids invaded freshwater habitats multiple times (figure 2). Our results suggest this might have happened exclusively via marginal marine settings, which likely allowed for the acquisition of adaptations for osmoregulation and desiccation resistance. Some eurypterids may have occupied both marine and marginal marine habitats, moving to the latter to breed or moult [2,33] but they are assigned to the habitat where they are preserved. However, the representation of marginal marine habitats as transitional stages to terrestrialization (as happens in freshwater crabs [72,73]) suggests a limited impact of such potential taphonomic bias. Eurypterids are interpreted as having a more arachnid-like mode of reproduction [74,75], which likely facilitated their transition to continental habitats as it eliminated the need to return to the sea to spawn [72]. We detect no association, however, between large body sizes and freshwater habitats (figure 3). Mycteropoids, which reached giant sizes in the Carboniferous, lived in swamps [31,58] where their thick cuticles may have facilitated osmoregulation [58] and protected them from desiccation. However, species of the eurypterine *Adelophthalmus*, which occupied similar habitats at this time and also evolved adaptations for terrestrial respiration, remained small [31].

The evolution of eurypterids prior to the Devonian biotic crisis was characterized by repeated acquisitions of large size accompanied by, but not dependent on, rare transitions to continental habitats (figure 2). Lineages that transitioned to freshwater environments remained low in diversity, while marine and marginal marine clades steadily diversified from the Ordovician to the late Silurian, at which time the diversity of eurypterids fell sharply in marine-influenced habitats. By the late Devonian, eurypterids were entirely continental, abandoning the niche of nektonic marine predators. This ecological shift reflects the freshwater radiation of stylonurine mycteropoids and eurypterine adelophthalmids following the Devonian biotic crisis, possibly owing to changes in freshwater ecosystems that previously constrained diversification. The increase in dissolved oxygen in the oceans in the Carboniferous [30] may have been paralleled in fresh water promoting an increase in eurypterid diversity in such settings (figure 4). The composition of freshwater fish communities shifted from placoderms to chondrichthyans and sarcopterygians at this time [76], perhaps changing the dynamics of their interactions with eurypterids. The diversity of these early freshwater eurypterids, however, was always limited, and there is no strong evidence for subsequent returns to marine environments. The incumbency of large predator nektonic fish in marine niches may have prevented Carboniferous eurypterid lineages from radiating back into marine habitats before declining towards extinction in the late Permian.

The niches occupied by mycteropids and hibbertopterids, which were bottom-dwelling sweep feeders [3,59,77] may have allowed these stylonurines to achieve large sizes by avoiding direct competition with gnathostomes such as *Megalichthys* and lungfish [76]. Large body sizes have been linked to higher extinction in vertebrates due in part to slower reproduction rates [78], although this relationship may be complex [79] and unreliable as a predictor for extinction risk in invertebrates [80]. Giant mycteropids may have survived the Devonian biodiversity crisis because, like other eurypterids, they were r-strategists and could reproduce quickly [2]. The morphology and trackways of later stylonurine eurypterids suggest they could tolerate terrestrial excursions [16,17,31] to seek more favourable conditions if necessary. Stylonurines retained large body sizes throughout their post-Devonian range, whereas the single surviving genus of eurypterines, *Adelophthalmus*, remained small. Thus, there was low variation in post Devonian eurypterid body size within the two clades, whether constrained to large (>50 cm) or small (<25 cm). A similar pattern of limited body size variation among closely related taxa was noted in transitions to freshwater among ray finned fishes [57].

### (c) Modes of eurypterid evolution

The evolution of giant size in eurypterids does not correlate with any single environmental driver, nor was it affected by ecological transitions or major extinction events (figure 3). Size ranges in other primarily aquatic arthropods, such as extant crustaceans, likewise do not show a strong extrinsic correlate [81] nor does size in fishes [82]. The multiple separate origins of large size in eurypterids are deeply nested in their respective clades (figure 2). In most cases, large size appeared ‘suddenly’ through dramatic jumps in morphospace, while closely related members of the same clades show little change, or even sustained stasis. This pattern is indicative of pulsed evolutionary processes [38], where sudden changes result in the exploration of new peaks in adaptive landscape [38,61,83]. Although these events might have been induced by environmental perturbations caused by as yet undetected factors, the lack of evidence for a clear driver of gigantism indicates that intrinsic factors may have played a larger role than previously recognized.

Rapid evolution followed by stasis is thought to be facilitated by r-selected reproductive strategies [84] characteristic of horseshoe crabs which spawn en masse [85]. R-strategists can respond more quickly to changes in the environment [86]. Such circumstances could result in multiple origins of giant size in response to events too rapid to leave a clear signal in the fossil record. Gigantism may also reflect genome size, which correlates with body size in some arthropods with determinate growth such as ostracods [87] and other crustaceans [88]. Growth in horseshoe crabs and arachnids is also determinate and they have experienced whole genome duplication events [89]. Horseshoe crabs exhibit independent marked increases in body size during the Carboniferous [90] and Jurassic [91]. Although these factors do not fully explain why eurypterids repeatedly attained sizes rarely matched by other arthropods, they may have facilitated the establishment of an evolutionary regime characterized by recurrent morphological jumps of large magnitude.

### (d) The nature of the data

It is a truism that the fossil record is incomplete, and this is inevitable in a clade that lacks a biomineralized cuticle and which invaded, later in its history, non-marine habitats that are not readily fossilized. The available sample is also subject to collection bias: most eurypterids are known from eastern North America and Europe and only a small number from the southern and eastern hemispheres [92] (electronic supplementary material, figure S8). The high diversity (~30 species) in a single well sampled formation with exceptional preservation, the late Silurian (Pridoli) Bertie Formation of upper New York State and southern Ontario, highlights how much diversity might be missing elsewhere. Preservation may favour smaller eurypterids [93]. The rarity of the largest individuals may compromise our knowledge of size ranges—the discovery of a large telson of *Eurypterus lacustris* more than 180 years after the species was established increased the known size range of this species by a factor of two [20]. The dominance of moults among eurypterid fossils [4] may further compromise estimates of maximum size as moults do not represent the largest size reached. Significant size differences between eurypterid sexes have not been reported [94] and may be overlooked: female horseshoe crabs (i.e. extant aquatic chelicerates) are larger than males [85,95].

### (e) Final conclusions

No single extrinsic factor explains the evolution of giant size in eurypterids. The clade diversified rapidly during the Silurian in marine environments, and colonized continental environments through marginal marine settings. Despite numerous transitions, and no clear reversals, successful continental radiations occurred only after the Devonian biotic crisis, indicative of a reorganization of ecological niches. Giant sizes evolved independently in numerous groups without obvious relation to habitat or environmental drivers such as temperature or oxygen levels. The pulsed nature of eurypterid body size evolution, potentially driven by intrinsic determinants, allowed them to evolve through an adaptive landscape with a lability unattained by other lineages of arthropods.

## Data Availability

The raw data used in this study are available for download from the electronic supplementary material in dataset 1. The data and code used in the analyses are available for download from Dryad [96]. Supplementary material is available online [97].
